# Identification and Analysis of Potential Immune-Related Biomarkers in Endometriosis

**DOI:** 10.1155/2023/2975581

**Published:** 2023-01-10

**Authors:** Yanan He, Jixin Li, Yanjun Qu, Liyuan Sun, Xibo Zhao, Han Wu, Guangmei Zhang

**Affiliations:** Department of Gynecology, The First Affiliated Hospital of Harbin Medical University, Harbin, Heilongjiang, China

## Abstract

**Background:**

Endometriosis is an inflammatory gynecological disease leading to deep pelvic pain, dyspareunia, and infertility. The pathophysiology of endometriosis is complex and depends on a variety of biological processes and pathways. Therefore, there is an urgent need to identify reliable biomarkers for early detection and accurate diagnosis to predict clinical outcomes and aid in the early intervention of endometriosis. We screened transcription factor- (TF-) immune-related gene (IRG) regulatory networks as potential biomarkers to reveal new molecular subgroups for the early diagnosis of endometriosis.

**Methods:**

To explore potential therapeutic targets for endometriosis, the Gene Expression Omnibus (GEO), Immunology Database and Analysis Portal (ImmPort), and TF databases were used to obtain data related to the recognition of differentially expressed genes (DEGs), differentially expressed IRGs (DEIRGs), and differentially expressed TFs (DETFs). Gene Ontology (GO) and Kyoto Encyclopedia of Genes and Genomes (KEGG) pathway enrichment analyses were performed on the DETFs and DEIRGs. Then, DETFs and DEIRGs were further validated in the external datasets of GSE51981 and GSE1230103. Then, we used quantitative real-time polymerase chain reaction (qRT-PCR) to verify the hub genes. Simultaneously, the Pearson correlation analysis and protein-protein interaction (PPI) analyses were used to indicate the potential mechanisms of TF-IRGs at the molecular level and obtain hub IRGs. Finally, the receiver operating characteristic (ROC) curve analysis was used to assess the diagnostic value of the hub IRGs.

**Results:**

We screened a total of 94 DETFs and 121 DEIRGs in endometriosis. Most downregulated DETFs showed decreased expression in the endometria of moderate/severe endometriosis patients. The top-ranked upregulated DEIRGs were upregulated in the endometra of infertile women. Functional analysis showed that DETFs and DEIRGs may be involved in the biological behaviors and pathways of endometriosis. The TF-IRG PPI network was successfully constructed. Compared with the control group, high C3, VCAM1, ITGB2, and C3AR1 expression had statistical significance in endometriosis among the hub DEIRGs. They also showed higher sensitivity and specificity by ROC analysis for the diagnosis of endometriosis. Finally, compared with controls, C3 and VCAM1 were highly expressed in endometriosis tissue samples. In addition, they also showed high specificity and sensitivity for diagnosing endometriosis.

**Conclusion:**

Overall, we discovered the TF-IRG regulatory network and analyzed 4 hub IRGs that were closely related to endometriosis, which contributes to the diagnosis of endometriosis. Additionally, we verified that DETFs or DEIRGs were associated with the clinicopathological features of endometriosis, and external datasets also confirmed the hub IRGs. Finally, C3 and VCAM1 were highly expressed in endometriosis tissue samples compared with controls and may be potential biomarkers of endometriosis, which are helpful for the early diagnosis of endometriosis.

## 1. Introduction

Endometriosis is an inflammatory gynecological disease characterized by the presence of endometrial tissues outside the uterus [1]. It affects approximately 10% of females in their reproductive years leading to a heavy financial burden on patients [2]. The typical clinical symptoms are chronic pelvic pain, dysmenorrhea, dyspareunia, and infertility, causing a decrease in patients' quality of life [3]. In addition, endometriosis surgery is the second most common surgery in premenopausal women. The occurrence and development of endometriosis are usually slow and are related to the local inflammatory response, proliferation, invasion, and angiogenesis of endometrial cells [4]. There are many theories about the etiology of endometriosis, but no exact theory can explain the pathogenesis of endometriosis [5]. Among the theories, the most prevalent is Sampson's theory of retrograde menstruation proposed in 1921. However, there are many arguments against this theory [6]. Because 90% of women have menstrual reflux, only 10% of women suffer from endometriosis. Although our understanding of endometriosis is growing, the exact molecular mechanisms underlying this tumor-like disease are still far from being understood. The pathophysiology of the occurrence and development of endometriosis is complex and depends on a variety of biological processes and pathways [3]. Therefore, there is an urgent need to determine reliable diagnostic biomarkers to predict early diagnosis and clinical severity.

The immune system plays a major role in survival in the pelvic microenvironment, including causing immune tolerance, depressing immunosurveillance, and escaping phagocytosis by immune cells [7]. Previous studies have indicated that immune-related genes (IRGs) play an important role in the complex regulatory network of tumors [8], and they have been explored to indicate the development of tumor immunity and the pathophysiological mechanisms of tumors [9], such as ovarian cancer. Emerging evidence has shown that women with endometriosis not only have a changed immune status of the endometrium but also have an altered peripheral immune system [10]. Consistent with the changes in the peritoneal environment of endometriosis, a large number of immune cells, inflammatory factors, and relevant cytokines have also been recruited to contribute to the abnormal immune environment in endometriosis [11, 12]. Nevertheless, the relationship between IRGs and the diagnosis of endometriosis patients is not clear, and further research is needed. This study is aimed at exploring the potential correlation between the onset of endometriosis and IRGs, which are potential molecular biomarkers to reveal new molecular subgroups for the early diagnosis of endometriosis.

Some transcription factors (TFs) are closely related to IRGs and can also regulate the function of IRGs in some diseases. Aberrant TF-IRGs could influence the various processes of tumor development. Additionally, the differential expression of TFs and their downstream target genes has been found to be related to the progression of endometriosis. Previous studies have shown that IRGs act as important regulators in diverse pathological processes. Therefore, studying the role of IRGs and their related molecular mechanisms in endometriosis is crucial, which is beneficial for exploring the pathogenesis of endometriosis and detecting more effective potential diagnostic markers.

## 2. Materials and Methods

### 2.1. Preparation and Processing of TF and IRG Data in Endometriosis

We searched two online TF datasets and downloaded 1665 TFs from the Human Transcription Factor Database (Human TFDB) [13] and 1639 TFs from the Human Transcription Factors Database [14]. The 1508 TFs obtained by the intersection of these two TF databases were used in our research on endometriosis. We constructed a diagnostic signature from the IRGs downloaded from the Immunology Database and Analysis Portal (ImmPort) database (http://www.immport.org) [15]. We used the Gene Expression Omnibus (GEO) database (https://www.ncbi.nlm.nih.gov/geo/) to analyze gene expression datasets. A total of 1871 series of endometriosis were retrieved from the database. We selected five GEO datasets (GSE7305, GSE7307, GSE51981, GSE1230103, and GSE23339) after filtering.

We matched the gene symbols of the data with the corresponding GEO platforms (GPL). In total, 10 endometriosis cases and 10 control samples were obtained from GSE7305, while 23 endometriosis patients' specimens and 18 control specimens were acquired from GSE7307. Both two expression microarrays were based on the GPL570 ((HG-U133_Plus_2) Affymetrix Human Genome U133 Plus 2.0 Array) platform. Moreover, GSE51981 and GSE120103, based on the GPL570 ((HG-U133_Plus_2) Affymetrix Human Genome U133 Plus 2.0 Array) and GPL6480 (Agilent-014850 Whole Huma Genome Microarray 4x44K G4112F) platform, respectively, were chosen for further validation. All of the data are freely available online.

### 2.2. Identification of DETFs and DEIRGs in Endometriosis

We selected two GEO gene expression datasets (GSE7305 and GSE7307) and divided the above data into the endometriosis group and the control group. First, the differentially expressed genes (DEGs) between the endometriosis and control samples were identified using the GEO2R online analysis tool (https://www.ncbi.nlm.nih.gov/geo/geo2r/), and the genes met the cutoff criteria based on the criteria of *P* < 0.05 and ∣log2FC | ≥1 [16]. Statistical analysis was carried out on each dataset, and the Venn diagram webtool (http://bioinformatics.psb.ugent.be/webtools/Venn/) was used to identify the intersection. The DEGs were then intersected with TFs from both databases and IRGs from the ImmPort database to obtain coupregulated differentially expressed TFs (DETFs), codownregulated DETFs, coupregulated differentially expressed IRGs (DEIRGs), and codownregulated DEIRGs.

### 2.3. GO Enrichment and KEGG Pathway Analyses of DETFs and DEIRGs

In this study, Gene Ontology (GO) enrichment and Kyoto Encyclopedia of Genes and Genomes (KEGG) pathway analyses of DETFs and DEIRGs were performed using the ClusterProfiler R package (version 3.18.0) [17], and *P* < 0.05 was considered statistically significant. The R package can automate the process of biological term classification and the enrichment analysis of gene clusters to unravel the biological meaning behind a large list of genes. GO analysis can be mainly classified into three domains: biological process (BP), molecular function (MF), and cellular component (CC). The version of R used in our research was 4.0.3.

### 2.4. PPI Network Construction and Hub Gene Identification

We used the Search Tool for the Retrieval of Interacting Genes (STRING) database (http://string-db.org/) to analyze protein-protein interaction (PPI) networks. It was essential to display the molecular mechanisms of key activities in endometriosis. To investigate the potential PPI relationships, the previously identified DETFs and DEIRGs were mapped to the STRING database. The PPIs were extracted with a combined score > 0.9. Subsequently, the visualized PPI network was constructed by Cytoscape software (version 3.7.1). The Hmisc R package (version 4.4.2) (https://hbiostat.org/R/doc/sintro.pdf) was utilized to test the correlations between DETFs and DEIRGs with the cutoff criteria set as correlation coefficient > 0.5 and *P* < 0.001. The Molecular Complex Detection (MCODE) plugin of Cytoscape software was utilized to recognize the most prominent clustering modules. Functional enrichment analysis of the genes in individual modules was achieved by DAVID, an online tool (https://david.ncifcrf.gov/), with a significance threshold of *P* < 0.05. Nodes with higher connectivity tend to be more important for maintaining the stability of the entire network. Therefore, cytoHubba, a plugin in Cytoscape, was used to screen out hub genes.

### 2.5. Collection of Human Tissues

Ectopic endometrium tissues were collected from chocolate cyst in endometriosis to identify hub genes expression (*n* = 12). Endometrium tissue from patients undergoing surgery for uterine fibroids served as a control group (*n* = 12). All patients in our study with or without endometriosis had no menstrual disorders. Patients who had received hormone therapy or other serious diseases were not included in this study. All tissue samples obtained were approved by the Ethics Commission of Harbin Medical University (202106).

### 2.6. Reverse Transcription and Quantitative Real-Time Polymerase Chain Reaction

12 endometriosis samples and 12 controls were frozen in liquid nitrogen, and total RNA was extracted using the TRIzol^®^ reagent (15596026, America). The relative expression of VCAM1, ITGB2, C3AR1, and C3 mRNA was normalized to GAPDH, and calculated using the 2^−ΔΔCt^ method (ΔCt = Ct^targetgene^ − Ct^internalcontrol^). The total RNA was used only if the A260/280 ratio of the absorbances was between 1.8 and 2.2 when measured by spectrophotometry. Reverse transcription was performed at 42°C (15 min) followed by 95°C (3 min), then in a 10 ml SYBR reaction system using the Talent qPCR PreMix (FP209-02, China) with 1 cycle of 95°C for 3 minutes, and 40 cycles of 95°C for 5 seconds and 60°C for 15 seconds. We identified the target mRNA sequences with ideal melting curves and sizes. Sequences of the primers are shown in Table 1.

### 2.7. Statistical Analysis

The method used to compare DETF expression in different severity groups of endometriosis was unpaired Student's *t* test. At the same time, the comparison method between the expression of DEIRGs in infertile and fertile endometriosis was unpaired Student's *t* test. The pROC R package (version 1.18.0) [18] was used to evaluate the sensitivity and specificity of DETFs and DEIRGs in the diagnosis of endometriosis. *P* < 0.05 was considered statistically significant in our study.

## 3. Results

### 3.1. Identification of DETFs and DEIRGs in Endometriosis

**Figure 1:**
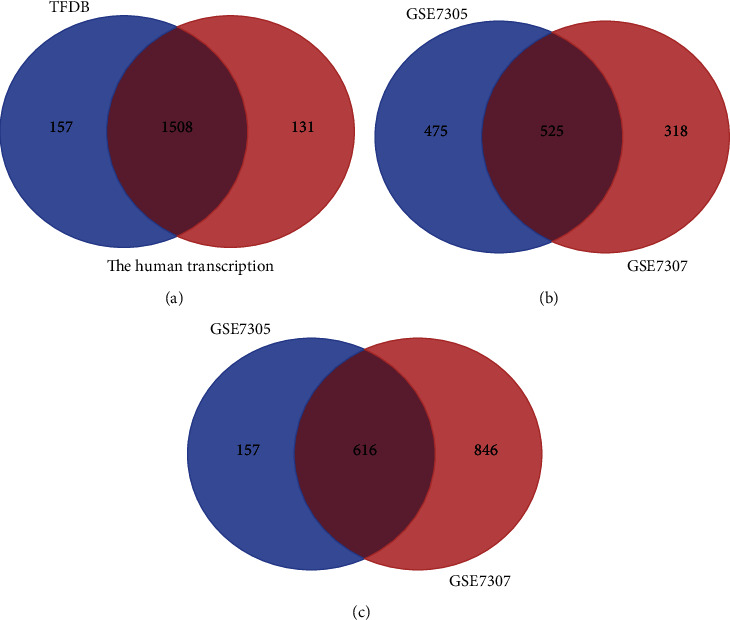
Identification of DETFs and 111 DEGs in endometriosis. (a) Identification of 1508 TFs from two TF databases (Human TFDB and The Human Transcription Factors). (b, c) Identification of 525 upregulated and 616 downregulated DEGs from GSE7305 and GSE7307.

**Figure 2:**
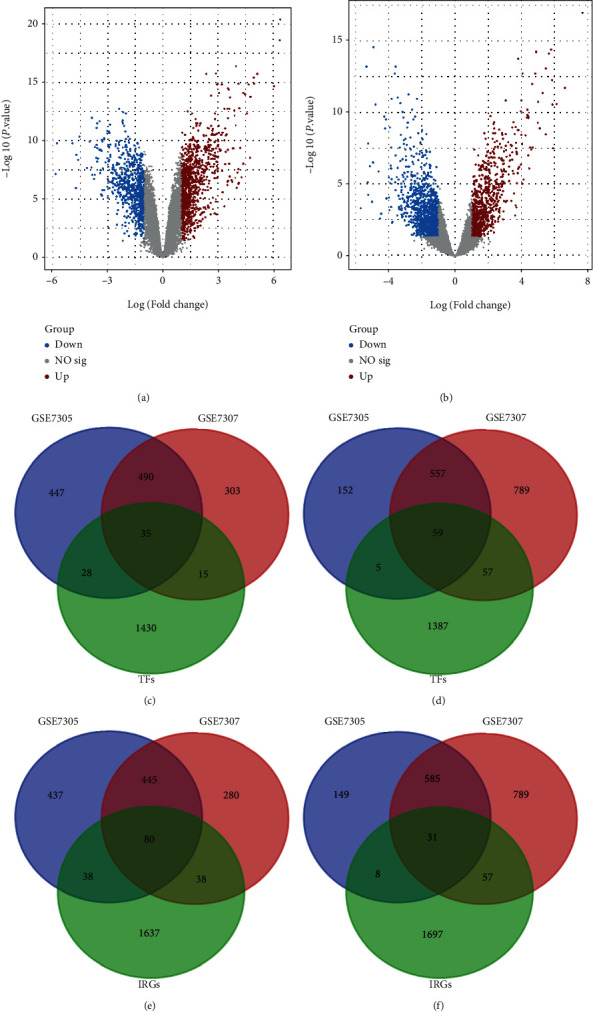
Identification of 94 DETFs and 111 DEIRGs in endometriosis. (a, b) Volcano plot of DEGs in GSE7305 and GSE7307. Red and blue data points represent upregulation and downregulation, respectively. (c, d) Identification of 35 upregulated and 59 downregulated DETFs from GSE7305, GSE7307 and TFs. (e, f) Identification of 80 upregulated and 31 downregulated DEIRGs from GSE7305, GSE7307, and IRGs.

**Figure 3:**
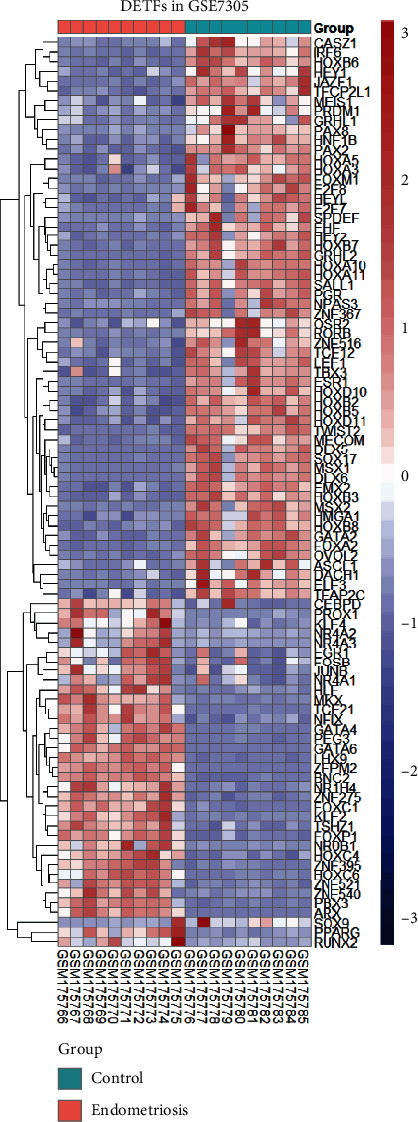
Heatmap of 94 DETFs in GSE7305. Each row represents a TF and each column represents a sample.

**Figure 4:**
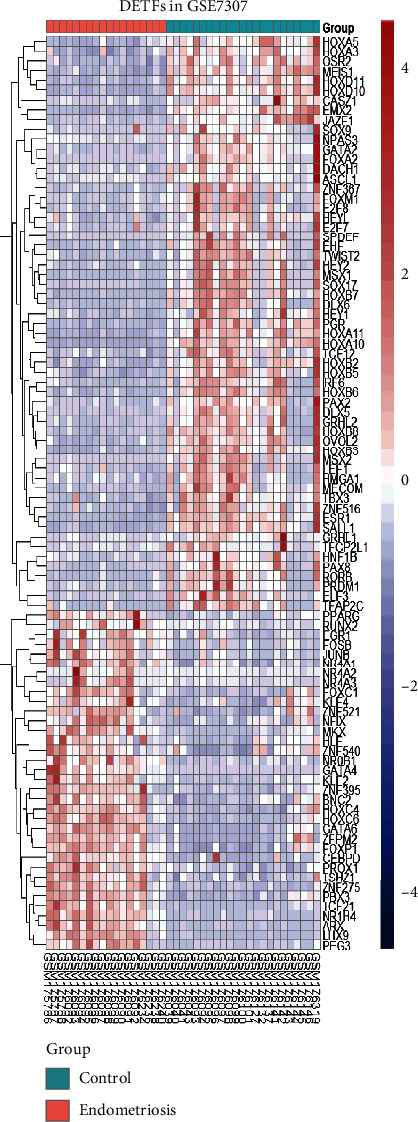
Heatmap of 94 DETFs in GSE7307. Each row represents a TF and each column represents a sample.

**Figure 5:**
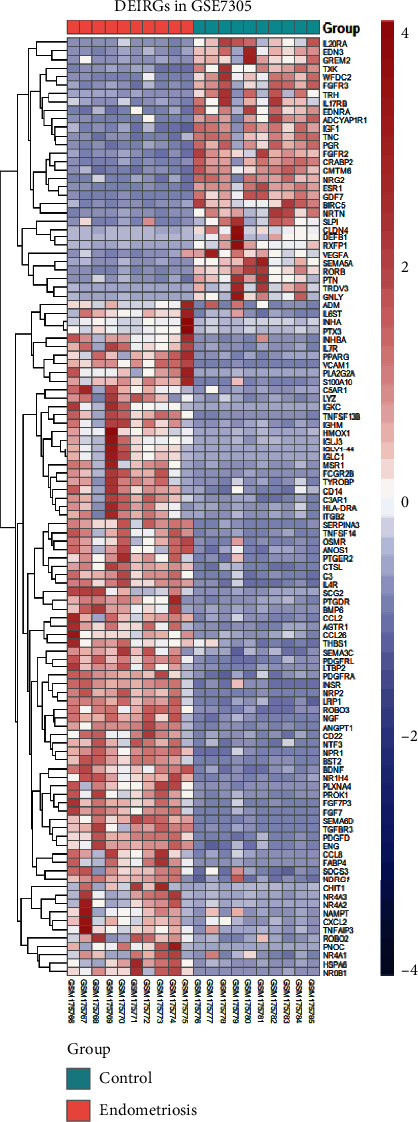
The heatmap of 111 DEIRGs in GSE7305. Each row represents an IRG.

**Figure 6:**
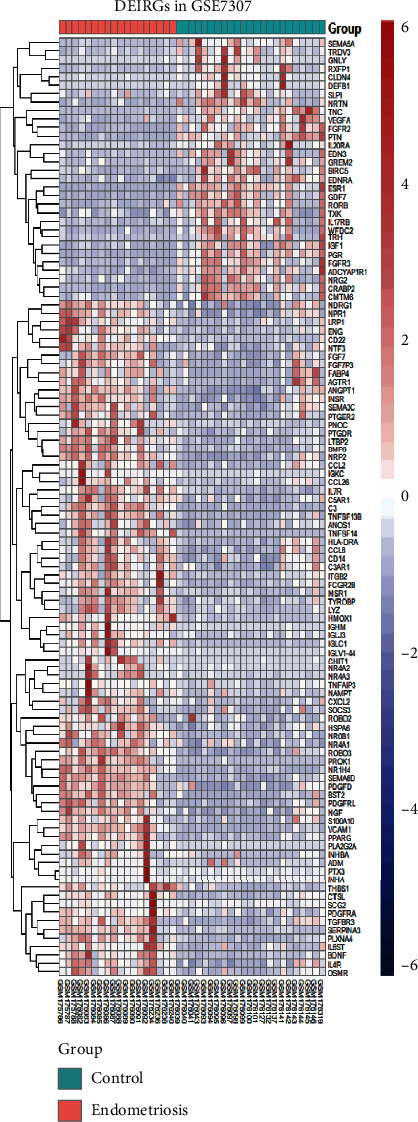
The heatmap of 111 DEIRGs in GSE7307. Each row represents an IRG.

We obtained 1508 intersecting TFs from Human TFDB and the Human Transcription Factors database (Figure 1(a)). Subsequently, we chose gene expression datasets from the GEO datasets. GSE7305 and GSE7307 were selected to identify DEGs because both included the endometrial samples with or without endometriosis. Based on the criteria of *P* < 0.05 and |log2FC| ≥1 [13], a total of 1141 DEGs from GSE7305 and GSE7307 were acquired by the GEO2R analysis tool, including 525 upregulated genes and 616 downregulated genes (Figures 1(b) and 1(c)). The DEGs were visualized by volcano plots in GSE7305 and GSE7307 (Figures 2(a) and 2(b)). Then, the results were intersected with 1508 TFs, identifying the 94 DETFs (35 upregulated DETFs and 59 downregulated DETFs) (Figures 2(c) and 2(d)). Similarly, 1793 unique IRGs were downloaded from the ImmPort database, and 111 DEIRGs (80 upregulated genes and 31 downregulated genes) were obtained from the intersection of the IRGs and the DEGs (Figures 2(e) and 2(f)). The DETFs and DEIRGs were visualized by a heatmap in GSE7305 and GSE7307, and there was a clear division between the endometriosis and control groups (Figures 3–6).

### 3.2. Validation of the DETFs in Different Severity Groups of Endometriosis

**Figure 7:**
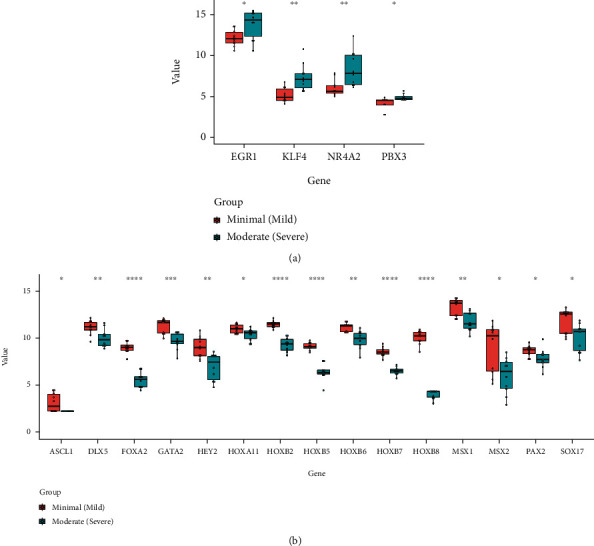
Expression of DETFs in different severity groups of endometriosis. (a) Upregulated DETFs were highly expressed in moderate/severe group. (b) Downregulated DETFs were decreased in the moderate/severe group (Unpaired Student's *t* test was used to compare two groups. ^∗^*P* < 0.05; ^∗∗^*P* < 0.01; ^∗∗∗^*P* < 0.001; ^∗∗∗∗^*P* < 0.0001).

**Figure 8:**
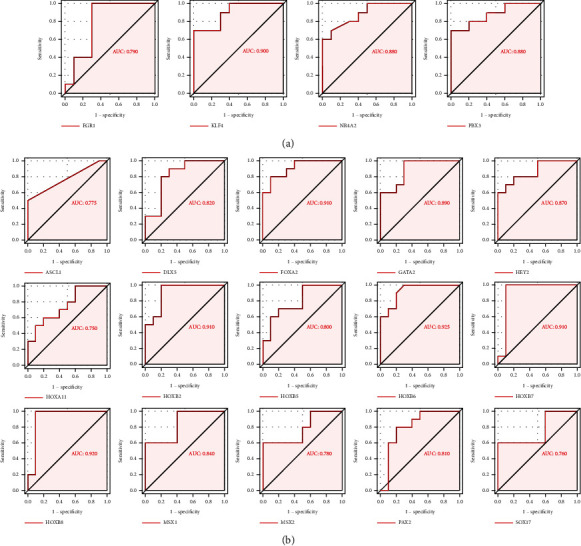
The ROC analysis of the hub IRGs predicting the severity of endometriosis. (a) ROC analysis of the upregulated DETFs predicting endometriosis severity in GSE51981. (b) ROC analysis of the downregulated DETFs predicting the severity of endometriosis in GSE51981.

To verify the credibility and applicability of the DETFs, we selected the external dataset GSE51981, which contained endometriosis samples with different severities. We obtained the 40 most highly expressed DETFs (14 upregulated DETFs and 26 downregulated DETFs) in 10 randomly selected samples from the minimal/mild group and moderate/severe group in GSE7305 and GSE7307. As shown in Figure 7(a), we found that a series of upregulated DETFs were still highly expressed in the moderate/severe group, and these upregulated DETFs could predict the severity of endometriosis by receiver operating characteristic (ROC) curve analysis (*P* < 0.05) (Figure 8(a)). Downregulated DETFs had a more pronounced advantage in predicting endometriosis severity than upregulated DETFs, and most of the downregulated DETFs remained expressed at low levels in the moderate/severe group (*P* < 0.05) (Figure 7(b)). In addition, the ROC curve provided powerful evidence to support this view, with area under the curve (AUC) values all over 0.7(Figure 8(b)).

### 3.3. The Expression of DEIRGs in Women with Endometriosis with or without Infertility

**Figure 9:**
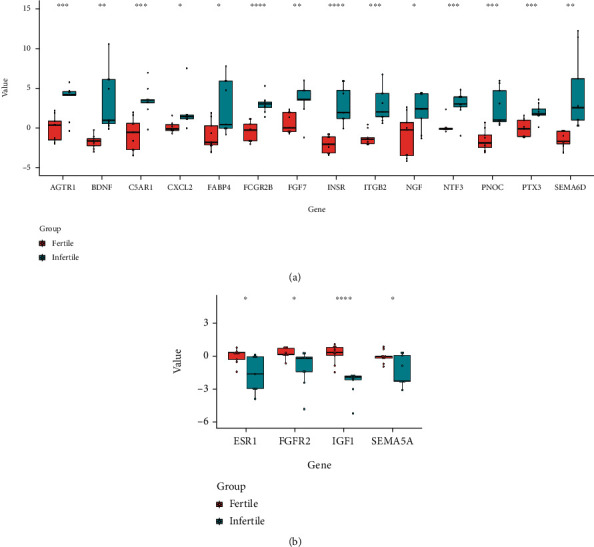
Expression of DEIRGs in the endometria of the infertile and fertile females with endometriosis. (a) Upregulated DEIRGs were highly expressed in the infertile group. (b) Downregulated DEIRGs were decreased in the infertile group (Unpaired Student's *t* test was used to compare two groups. ^∗^*P* < 0.05; ^∗∗^*P* < 0.01; ^∗∗∗^*P* < 0.001; ^∗∗∗∗^*P* < 0.0001).

**Figure 10:**
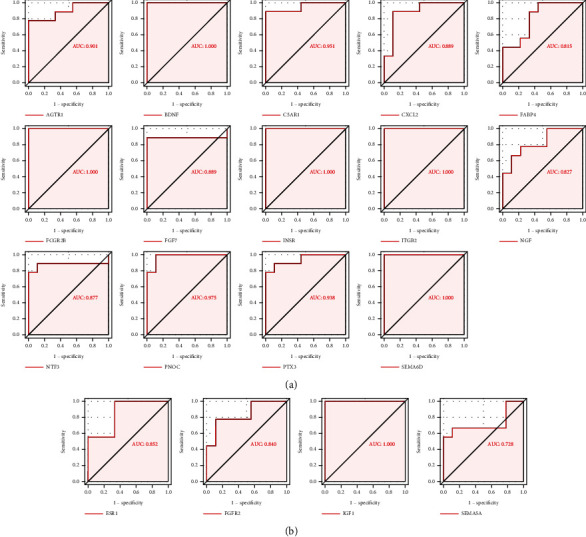
The ROC analysis of the hub IRGs predicting the infertility of endometriosis. (a) The ROC analysis of upregulated DEIRGs predicting infertility of endometriosis in GSE120103. (b) The ROC analysis of downregulated DEIRGs predicting infertility of endometriosis in GSE120103.

The GSE120103 dataset was chosen for subsequent validation because it included infertile and fertile females with endometriosis, and we obtained the top 40 DEIRGs expressed in it. Interestingly, most of the upregulated DEIRGs were increased in infertile females with endometriosis, while some downregulated DEIRGs were expressed at low levels in the endometria of infertile women (*P* < 0.05) (Figures 9(a) and 9(b)). For this result, we conducted ROC analysis to evaluate the values of DEIRGs in the diagnosis of endometriosis, and the AUC also verified the sensitivity and specificity of DEIRGs with *P* < 0.05 (Figures 10(a) and 10(b)).

### 3.4. Functional Enrichment Analysis of DETFs and DEIRGs

**Figure 11:**
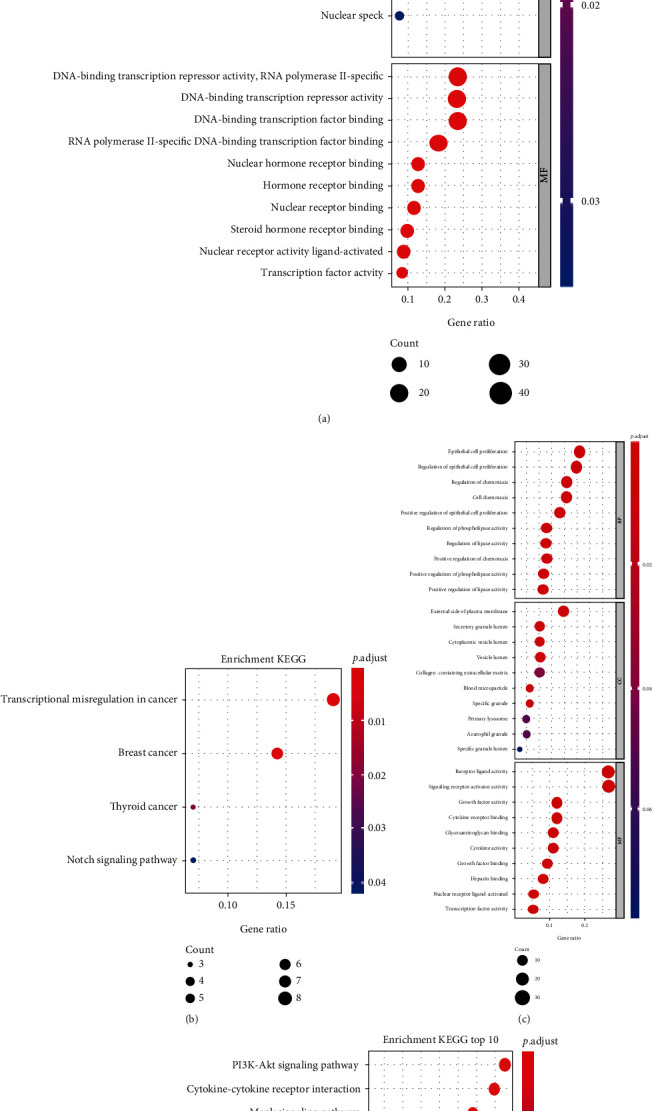
GO and KEGG pathway analyses of 94 DETFs and 111 DEIRGs in endometriosis. (a) The GO analysis of DETFs. (b) The KEGG analysis of DETFs. (c) The GO analysis of DEIRGs. (d) The KEGG analysis of DEIRGs. The GO analysis included the biological processes, cellular components, and molecular functions (*P* < 0.05).

To indicate the biological properties of DETFs and DEIRGs, functional analysis was performed, including GO functional and KEGG encrichment analyses. The enriched GO terms were divided into BP, CC, and MF ontologies. The GO functional enrichment results of the DETFs were mainly enriched in the BP ontology. BP analysis showed that the DETFs were significantly enriched in reproductive structure or system development, cell fate commitment, and anterior/posterior pattern specification. For the cell component, the DETFs were enriched in the transcription regulator complex and nuclear speck RNA polymerase II transcription regulator complex. The MF ontology of DETFs was mainly related to ligand-activated transcription factor activity, RNA polymerase II-specific DNA-binding transcription factor binding, and nuclear receptor binding or activity (Figure 11(a)). For the KEGG analysis, the DETFs were mainly enriched in the signaling pathways associated with transcriptional misregulation in cancer and the Notch signaling pathway (Figure 11(b)).

Likewise, the DEIRGs were also enriched in the regulation chemotaxis, lipase or phospholipase activity, cytoplasmic vesicle lumen, external side of plasma membrane, cytokine activity, and nuclear receptor activity (Figure 11(c)). The KEGG pathways of the DEIRGs were mainly enriched in viral protein interactions with cytokines and cytokine receptors, the PI3K-Akt signaling pathway, and the MAPK signaling pathway (Figure 11(d)).

### 3.5. PPI Network Construction and Pearson's Correlation Analysis

**Figure 12:**
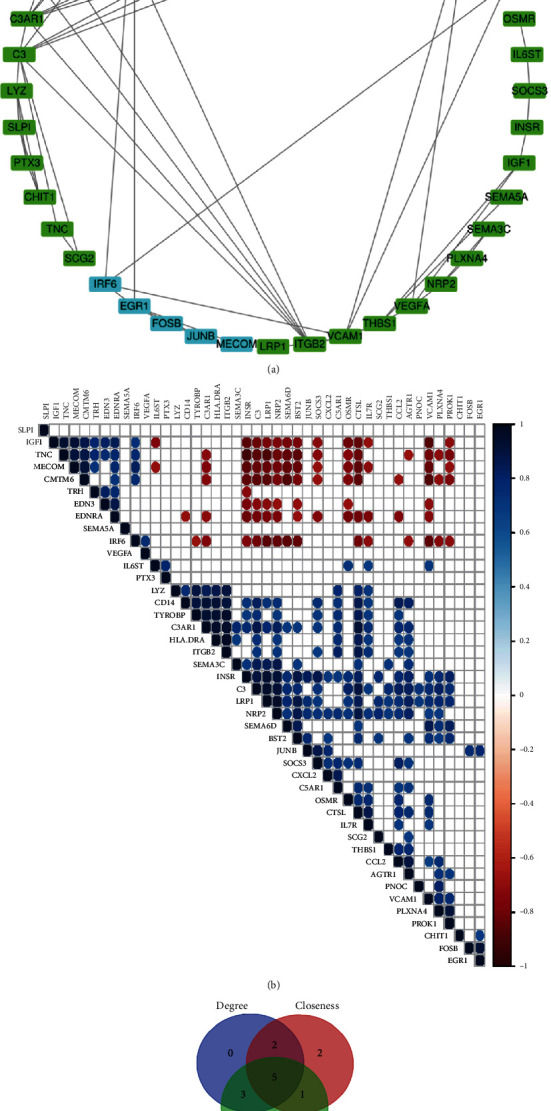
PPI network, modules, and identification of 5 hub genes. (a) The PPI network of the 94 DETFs and 111 DEIRGs (interaction score > 0.9). Blue nodes represent transcription factors, and green nodes represent immune-related genes. (b) The correlation of DETFs and DEIRGs (*P* < 0.001). (c) Venn diagram showing the overlap of the top ten nodes in topological features of degree, betweenness, and closeness.

**Figure 13:**
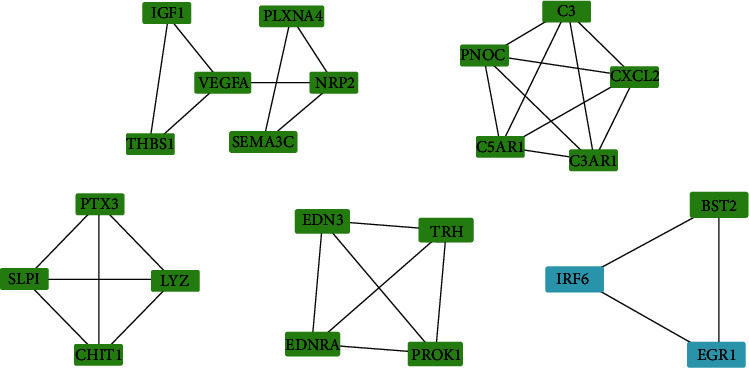
Modules 1-5 from the PPI network of TFs-IRGs.

Protein interactions between the DETFs and DEIRGs were constructed using the STRING online database, and the PPI network was constructed using Cytoscape. Five subnetworks were recognized. Therefore, we obtained TF-IRG regulatory networks containing 44 nodes and 73 edges to accurately illustrate the regulatory relationships between the DETFs and DEIRGs (Figure 12(a)). Pearson's correlation analysis was used to analyze the TF-IRG regulatory network, and most nodes were correlated with other nodes at the expression level with *P* < 0.001 (Figure 12(b)). The MCODE plugin of Cytoscape was used to complement the module analysis, with the corresponding modules shown in Figure 13. Furthermore, the most significantly enriched functional modules were those related to complement and coagulation cascades, *Staphylococcus aureus* infection, proteoglycans in cancer, focal adhesion, and the Rap1 signaling pathway (Table 2).

Subsequently, we used the cytoHubba plugin of Cytoscape to identify hub genes according to the three most important topological features in network analysis, including degree, betweenness, and closeness. We then ranked the top ten nodes for each set of the three different topological measurements (Table 3). As a result, we obtained five nodes (CXCL2, C3, VCAM1, ITGB2, and C3AR1) in all three of the lists (Figure 12(c)). These five DEIRGs can therefore be considered hub genes in the regulatory network.

### 3.6. Identification and Validation of Hub IRGs

**Figure 14:**
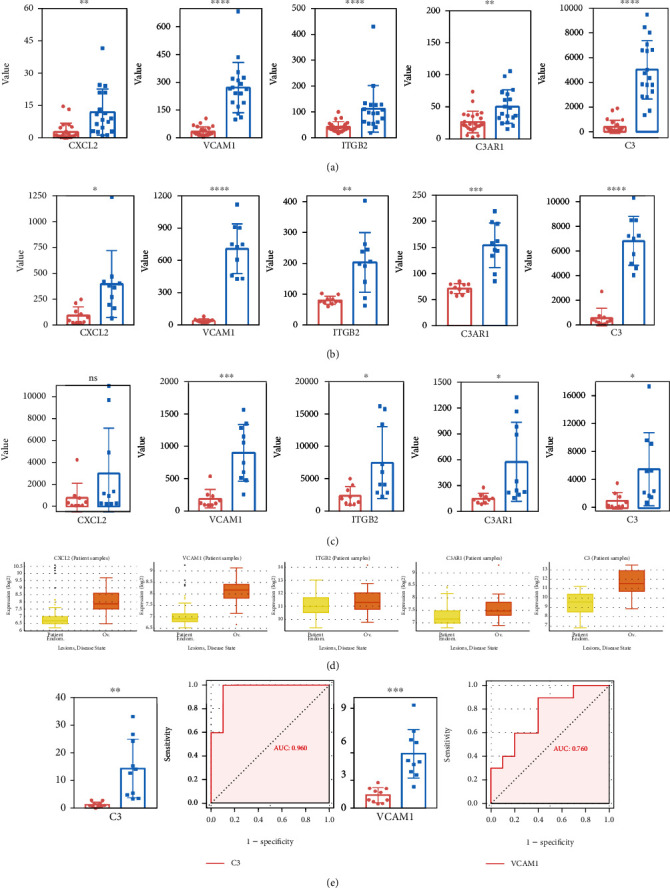
Analysis and validation of the differentially expressed hub IRGs in four databases. (a) GSE7305 dataset. (b) GSE7307 dataset. (c) Validation in the GSE23339 dataset. (d) Validation in the publicly accessible ENDOMET Turku Endometriosis Database. (e) The differences in the mRNA expression levels of C3 and VCAM1 by qRT-PCR between the endometriosis tissues and the controls and the ROC analysis. (a, b) C3. (c, d) VCAM1 (Unpaired Student's *t* test was used to compare two groups. ^∗^*P* < 0.05; ^∗∗^*P* < 0.01; ^∗∗∗^*P* < 0.001; ^∗∗∗∗^*P* < 0.0001).

**Figure 15:**
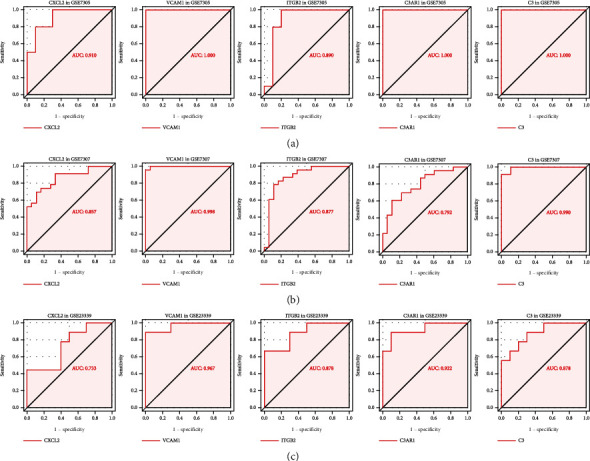
The ROC analysis of the hub IRGs predicting the onset of endometriosis. (a) GSE7305 dataset. (b) GSE7307 dataset. (c) GSE23339 dataset.

As shown in GSE7305 and GSE7307, the expression of each hub IRG was significantly higher in the endometriosis group than in the control group in the box plot (*P* < 0.05) (Figures 14(a) and 14(b)). The GSE23339 dataset (GPL6102 Illumina human-6 v2.0 expression beadchip) and the publicly accessible ENDOMET Turku Endometriosis Database were also used to verify the DEIRGs (Figures 14(c) and 14(d)). However, in the additional database validation of GSE23339, CXCL2 was not statistically significant. Thus, four hub genes were obtained (C3, VCAM1, ITGB2, and C3AR1). In view of the above results, we verified the above four target genes by qRT-PCR, and the highly expressed C3 and VCAM1 were statistically significant in endometriosis, and the AUCs were 0.96 and 0.76 (Figure 14(e)). However, ITGB2 and C3AR1 were not statistically significant (Figure S1). The AUCs calculated from ROC analysis in GSE7305 (Figure 15(a)), GSE7307 (Figure 15(b)), and GSE23339 (Figure 15(c)) were used to evaluate the diagnostic value of endometriosis. The AUC values of all four hub DEIRGs were over 80%, which meant that the hub DEIRGs played a critical role as novel biomarkers for endometriosis.

## 4. Discussion

Endometriosis is a common benign gynecological disorder characterized by immunity, inflammation, and hormone dependence. Previous studies on endometriosis have mainly focused on TFs [19] or IRGs [20], and there have been few studies on TF-related IRGs in endometriosis. TF-related IRGs not only function in immunity regulation but can also be used as prognostic biomarkers and play a key role in the development of cancer [21]. The exact molecular mechanisms of endometriosis are still unclear, and the current treatments are limited. Therefore, the discovery of new therapeutic targets and potential diagnostic biomarkers remains a research focus.

With the rapid development of the high-throughput methods and data analysis of various databases, Bohler et al. have focused on bioinformatics analysis, which can also serve as the basis for molecular biology experiments of endometriosis [22]. This study mainly analyzed DETFs and DEIRGs in endometriosis by bioinformatics methods and analyzed the expression of DETFs and DEIRGs in the endometria of women with different disease severities and infertility statuses. In addition, the enrichment analysis and networks were performed and constructed on DETFs and DEIRGs to discover valuable TFs, IRGs, and related endometriosis pathways.

Among patients with different severities of endometriosis, upregulated DETFs were more highly expressed in patients with moderate/severe endometriosis. However, downregulated DETFs presented the opposite trend. We believe that this phenomenon may be related to the degree of macrophage infiltration of different severities of endometriosis. Compared with the r-AFS stage I-II of endometriosis, the proportion of M2 macrophages in stage III-IV endometriosis was higher, suggesting that the degree of M2 macrophage infiltration was related to the severity of endometriosis [23]. M2 macrophages have anti-inflammatory properties, promote wound healing, promote fibrosis, and enable the immune escape of ectopic endometrium [24]. In addition, M2 macrophages produce matrix metalloproteinases (MMPs), such as MMP 9, which promote the ectopic growth and progression of endometrial cells by degrading the extracellular matrix and enhancing intercellular adhesion [25, 26]. At the same time, the results of DETF enrichment analysis also suggested that they may affect the activation of macrophages in endometriosis, such as the Notch pathway, which was consistent with the findings of previous research [27].

In fertile or infertile women with endometriosis, most of the upregulated DEIRGs were also highly expressed in infertile women, whereas only 4 downregulated DEIRGs were significantly expressed at a low level in the infertile group. Immunity, inflammation, and DEGs have important implications in infertile patients. These factors may affect the expression of IRGs, and that they and IRGs coregulate to influence susceptibility in patients with endometriosis-related infertility. A previous study found that BDNF (Met) single-nucleotide polymorphism, an IRG, was associated with endometriosis-related infertility women, suggesting that low levels of BDNF may be responsible for poor in vitro fertilization (IVF) outcomes in infertile patients with the BDNF (Met/Met) genotype [28]. Yin et al. studied another IRG, PTX3, which is also associated with endometriosis-related infertility [29]. In our study, IGF1 had lower expression levels in infertile women with endometriosis, and it had the ability to attenuate oocyte and embryo development resulting in endometriotic infertility, as reported in the study of Ding et al. [30]. At the same time, ESR1 showed low expression, and related studies suggested that ESR1 can affect the possibility of pregnancy in infertile patients with endometriosis [31].

In this study, the GO and KEGG enrichment analyses showed that the IRGs were mainly related to immune-related functions and pathways, such as the external side of the plasma membrane, cytokine activity and nuclear receptor activity, the PI3K-Akt signaling pathway, and the MAPK signaling pathway. In general, the external side of the plasma membrane plays an important role in endometriosis immunity. Antigens derived from the plasma membrane might directly assay reactive autoantibodies to indicate the immunoreactivity of endometriosis severity [32]. Cytokine activity, such as proinflammatory cytokines (IL-1*β*, IL-6) and tumor growth factor-beta (TNF-*β*), plays an important role in evading immune surveillance and predicting the disease severity of endometriosis [33, 34]. In addition, upregulation of MAPK subfamilies promoted the occurrence of endometriosis by influencing the function of various cytokines, including IL-6 and IL-8 [35]. The PI3K-Akt and MAPK pathways are interconnected with each other [36, 37]. The activation of the PI3K-Akt signaling pathway and the ERK-related intracellular MAPK signaling pathway was correlated with endometriosis [36], and both were shown to be involved in the immunity [38]. In addition, our research further identified the involvement of IRGs in the regulation of the PI3K-Akt signaling pathway and MAPK signaling pathway in endometriosis. A previous study showed that upregulation of the adaptor protein SHC1 had the ability to activate the PI3K-Akt and/or MAPK pathways in endometriosis samples [39]. The activation of the PI3K-Akt and MAPK pathways was associated with the immune-related pathway, nuclear factor-*κ*B (NF-*κ*B) signaling pathway in endometriosis cells [40]. Therefore, the study of IRGs in endometriosis is essential. These findings shed light on the screening of new potential biomarkers and the early diagnosis of endometriosis.

A previous study reported a TF-targeted gene network indicating the onset of endometriosis [41]. To further investigate the possible underlying molecular regulatory mechanisms, a TF-IRG network was constructed to study the mechanism of endometriosis. A total of 39 IRGs (RND3, PLK2, AURKA, RCAN1, EZH2, etc.) were selected to analyze the TF-IRG PPI network. Five DETFs (IRF6, EGR1, FOSB, JUNB, and MECOM) were connected with several IRGs. The genes in the PPI network were closely linked and cross regulated with each other. For instance, in the multifunctional network, IRF6/BST2 was involved in the regulation of immunity. Currently, there are many related studies on IRF6 and BST2 in immunity. Aberrant DNA methylation of IRF6 and BST2 in CD4+ T cells induced autoimmune responses [42]. Meanwhile, Figure 13 shows the predicted binding sites of IRF6 and BST2, which suggests that IRF6 and BST2 may function through mutual regulation. In a human papillomavirus type 16 (HPV16) study of host immunity, inhibition of IRF6 was responsible for immune escape from HPV16 blocking IL-1*β* secretion [43]. In this study, we found that BST2/CD317 in combination with TLR agonists specifically presented Ag by plasmacytoid dendritic cells in vivo, which contributed to the strong cellular and humoral immune responses [44]. However, the detailed mechanism of the PPI network should be elucidated in the future. Our findings provide an informatics basis for future research in this direction.

In this research, we mainly aimed to construct an IRG-related diagnostic model, which was established based on DEGs. ROC analysis revealed that four IRGs can be used as potential biomarkers of endometriosis, which also demonstrated the feasibility in terms of the AUC, a signal for endometriosis occurrence. Recently, C3 was considered a candidate diagnostic biomarker of endometrosis, and its expression was correlated with the engraftment of the endometriotic cysts [45]. The overexpression of VCAM-1 on the peritoneum of endometriosis had been revealed by Schutt et al. [46]. The increased expression of ITGB2 had been previously reported in endometriosis tissues compared with normal tissues [47], and high C3AR1 expression might be used as a diagnostic factor for the endometriosis-associated malignant phenotype [5]. In this research, two hub DEIRGs (C3 and VCAM1) with diagnostic value were obtained. However, this research had some limitations. First, the applicability of the diagnostic model needs to be validated in a larger sample population in future studies. Second, we will continue to complete the molecular mechanism study on the role of IRGs in endometriosis.

## 5. Conclusion

The TF-IRG network could be used to present novel prospective molecular mechanisms underlying the development of tumors [45]. However, studies of the regulatory mechanisms underlying TFs and IRGs in endometriosis are still in progress. In our study, IRGs were used to construct a diagnostic model to predict the onset of endometriosis patients by bioinformatics analysis. ROC analysis confirmed that the diagnostic value of hub genes (C3 and VCAM1) was clinically feasible. Additionally, the TF-IRG regulatory network broadened the horizon for research concerning the pathogenesis of endometriosis.

## Figures and Tables

**Table 1:** Sequences of the primers used for quantitative real-time polymerase chain reaction.

Gene	Forward primer (5′-3′)	Reverse primer (5′-3′)
VCAM1	GGAGCTCTACTCATTCCCTAGA	CTAGGAACCTTGCAGCTTACA
ITGB2	GTGACCAGTAGGATGGTGAAG	GACCCTGGAGGAGAGTTTATTG
G3AR1	GAAACCAGCCCACTGGATAA	TGGTAGCTCAGACTCGTAGAA
C3	ACGGCCTTTGTTCTCATCTC	CAAGGAAGTCTCCTGCTTTAGT
GAPDH	CTGGGCTACACTGAGCACC	AAGTGGTCGTTGAGGGCAATG

**Table 2:** Module analysis of the protein-protein interaction network.

Pathway description	FDR	Nodes	Genes
Staphylococcus aureus infection	2.36*E* − 03	3	C3, C5AR1, and C3AR1
Complement and coagulation cascades	2.36*E* − 03	3	C3, C5AR1, and C3AR1
Proteoglycans in cancer	2.82*E* − 02	3	IGF1, THBS1, and VEGFA
Focal adhesion	2.82*E* − 02	3	IGF1, THBS1, and VEGFA
Rap1 signaling pathway	2.82*E* − 02	3	IGF1, THBS1, and VEGFA

**Table 3:** Top ten nodes in topological analyses of degree, betweenness, and closeness.

Degree	Closeness	Betweenness
AGTR1	C3AR1	C3
C3	ITGB2	VCAM1
C3AR1	C3	ITGB2
ITGB2	VCAM1	AGTR1
PNOC	CXCL2	VEGFA
C5AR1	CCL2	C3AR1
EGR1	PNOC	THBS1
CXCL2	C5AR1	CCL2
VCAM1	BST2	IGF1
VEGFA	CMTM6	CXCL2

## Data Availability

All of the data we used in this study were publicly available as described in the methods section and can be found in online Github page: https://github.com/zgm19661026/zgm19661026.git.

## Supplementary Materials

Supplementary Materials Figure S1: the differences in the mRNA expression levels of ITGB2 and C3AR1 by qRT-PCR between the endometriosis tissues and the controls. (A) ITGB2. (B) C3AR1.
