# Supplementary figures and images for: Identification and Analysis of Potential Immune-Related Biomarkers in Endometriosis
Source: J Immunol Res. 2023 Jan 10;2023:2975581. doi: 10.1155/2023/2975581 (PMC9845045; doi:10.1155/2023/2975581)

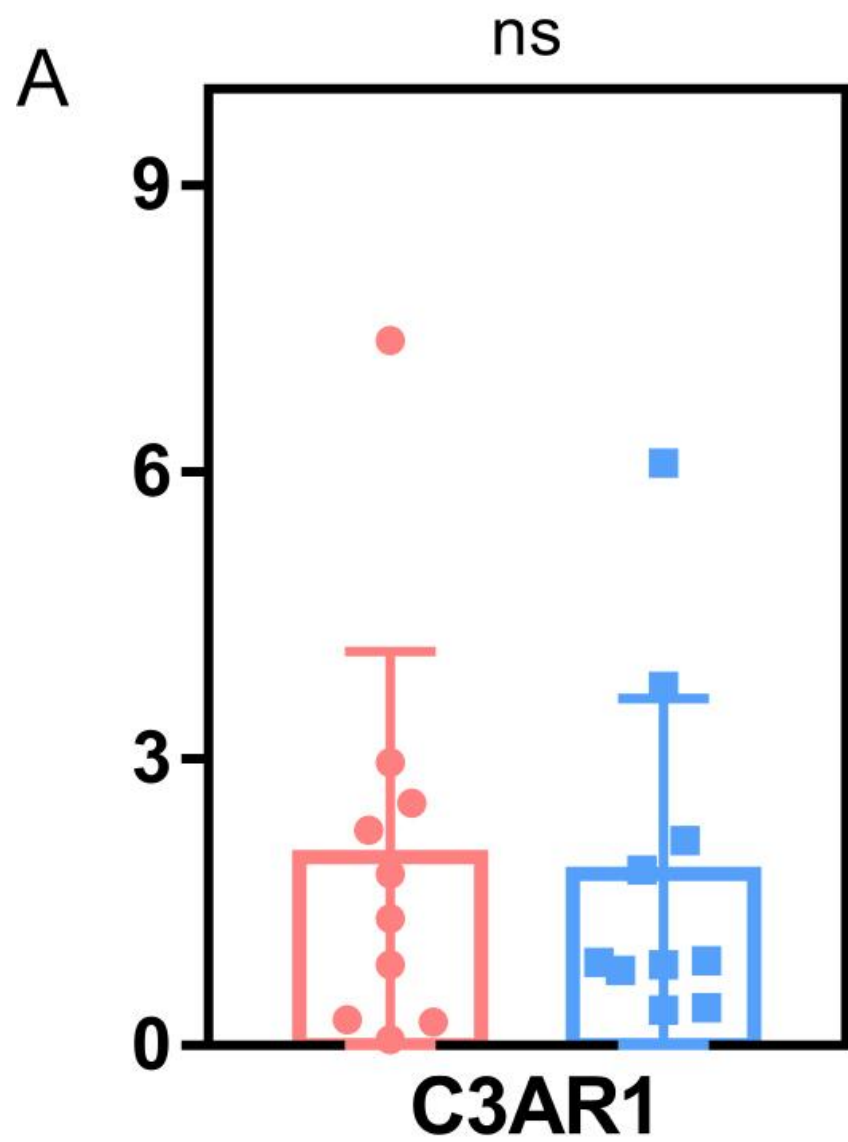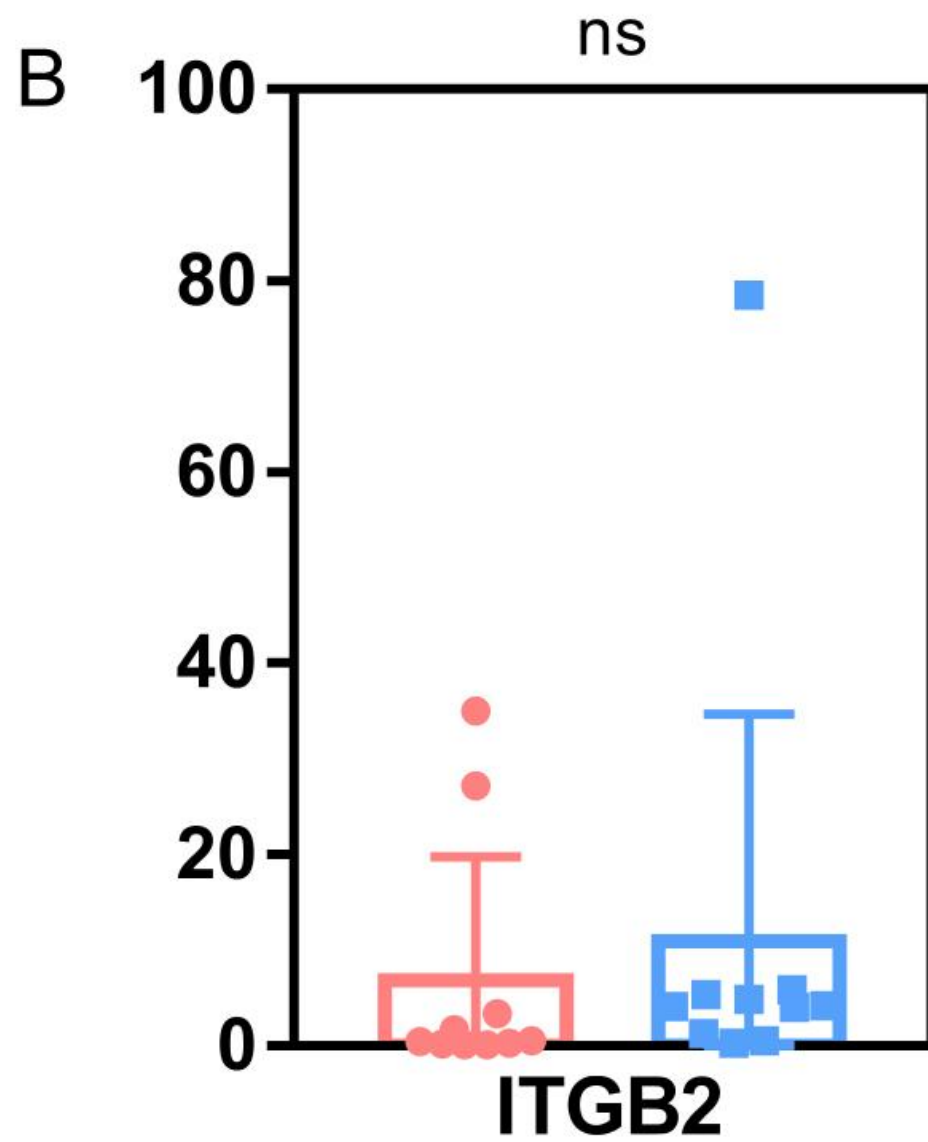

Supplement: Supplementary Materials — Figure S1: the differences in the mRNA expression levels of ITGB2 and C3AR1 by qRT-PCR between the endometriosis tissues and the controls. (A) ITGB2. (B) C3AR1. [file 2975581.f1.pdf]
